# Breast Milk Pasteurization: Appropriate Assays to Detect HIV Inactivation

**DOI:** 10.1155/IDOG/2006/95938

**Published:** 2006-01-25

**Authors:** Caroline J. Chantry, Barbara F. Abrams, Richard M. Donovan, Kiersten A. Israel-Ballard, Haynes W. Sheppard

**Affiliations:** ^1^Department of Pediatrics, University of California, Davis Medical Center, 2516 Stockton Boulevard, Room 334, Sacramento, CA 95817, USA; ^2^Division of Epidemiology, School of Public Health, University of California, 140 Earl Warren Hall #7360, Berkeley, CA 94720-7360, USA; ^3^California Department of Health Services, Viral and Rickettsial Disease Laboratory, 850 Marina Bay Parkway, Richmond, CA 94804, USA

We read the recent article “Breast milk pasteurization in developed countries to reduce HIV transmission. Do the benefits outweigh the risks?” in *Infectious Diseases in Obstetrics and Gynecology* with great concern. The authors tested two paired specimens of heated and unheated breast milk using HIV RNA quantification (NASBA and Roche ULTRA PCR). They found no decrease in HIV RNA levels between the heated and unheated samples and thus concluded there to be insufficient data to recommend heat treatment as a safe alternative in resource-rich countries. We are particularly concerned that they have misinterpreted their results and that this confusion may be perpetuated in discussions and policies around the globe, most importantly in resource-poor regions.

We strongly encourage the authors, editors, and readership of your journal to reinterpret the results presented since the HIV detection method used by Giles and Mijch does not differentiate between active (infectious) and inactive (noninfectious) HIV in breast milk. Our team has been investigating the safety of heat-treated breast milk as an infant feeding option for mothers in developing countries and have recently published the results of pilot safety data [2]. We have designed a simple “flash-heat” treatment method that a mother could use in her home or over a fire, similar to commercial high-temperature, short-time (HTST) pasteurization. Although the study published by Chantry et al [3] used a similar heating method and demonstrated destruction of HIV proviral DNA in HIV-infected breast milk cells [3], the method used in that report achieved higher milk temperatures due to smaller milk volumes and Pyrex glass. In designing a more gentle heating method, we also found, as reported by Giles and Mijch, no decrease in HIV RNA.

We ascertained, however, that *assaying for presence of viral RNA*, as performed by Giles in the above article, is *not an adequate technique for detecting infectious virus in heated breast milk*. Nucleic acid is very resistant to heating, up to the boiling point of water. It is to be expected that viral nucleic acid will remain postheating and will be detected by PCR-based assays even after the virus itself is rendered totally incapable of replication due to destruction of vital enzyme activities, structural proteins, and membrane structures due to the heat. We acknowledge that RNA detection is commonly used for quantification of HIV in both plasma and unheated breast milk. In order to determine the effect of heat on HIV in breast milk, however, the assay must effectively distinguish between live versus inactivated virus. We have demonstrated this in our recent pilot work comparing the flash-heat method with Pretoria pasteurization, another simple technique mentioned by Giles and Mijch [4, 5] . We found no decrease in cell-free HIV RNA as determined by TaqMan Real-time RNA PCR in breast milk (Log HIV RNA (SD) in unheated milk = 8.00(0.03) versus heated milk = 7.95(0.03)). We recognized the need for an alternative assay to accurately assess virus viability and, as traditional coculture methods are difficult with breast milk due to the innate antiviral properties of the milk, we chose quantitative measurement of reverse transcriptase (RT) as a marker for viable HIV (ExaVir Quantitative Reverse Transcriptase Load Kit, Cavidi, Uppsala Sweden). In contrast to our TaqMan PCR data from the same samples, we found inactivation of ≥ 3 logs of HIV-1 as detected by enzymatic activity of RT in postheated samples, with the flash-heat method more effectively eliminating RT than Pretoria pasteurization. We have subsequently confirmed these results by directly assaying for infectivity using transactivation of a green fluorescent protein (GFP) reporter group (data unpublished). Although we acknowledge that detection of HIV activity in breast milk can be challenging, we would encourage the authors to repeat their work using an appropriate assay.

We recognize the concerns mentioned by the authors regarding the impact of heat on vitamins, proteins, immunoglobulins, and the antimicrobial properties of breast milk. Low-temperature, long-time (LTLT) heat treatments, for example, Holder pasteurization, typically preserve nutrients less than HTST methods do. We reported pilot data suggesting limited impact on vitamins and proteins using flash-heat [2]. Our ongoing study is investigating the above concerns in-depth and we hope to have this data available in the near future.

We agree that it is not currently justifiable to recommend heat treatment of HIV-infected breast milk in resource-rich countries. However, we are concerned that the results, presented by Giles and Mijch of two heated breast milk samples demonstrating persistent HIV RNA being interpreted as “persistent HIV” without further exploration of viral infectivity, may have unwarranted repercussions. We strongly urge re-consideration of the results in light of our findings that HIV RNA is detectable after heating with no demonstrable activity of RT, which is necessary for virus to replicate. Heat treatment of breast milk is a recognized infant feeding option by the World Health Organization for HIV positive mothers who live in areas where no other alternatives are available. While we acknowledge that further research is needed, caution should be used when stating conclusions that may negatively impact policy makers' decisions regarding what may be appropriate in such communities.

## signatures

Caroline J. Chantry Barbara F. Abrams Richard M. Donovan Kiersten A. Israel-Ballard Haynes W. Sheppard
